# Hospital‐to‐Home Transitions for Lung Cancer Patients—A Qualitative Study of Healthcare Professionals' Experiences

**DOI:** 10.1002/nop2.70143

**Published:** 2025-02-07

**Authors:** Charlotte Nilsen, Trine Oksholm, Gudmund Ågotnes, Gro Beate Samdal, Sidsel Ellingsen

**Affiliations:** ^1^ Centre of Diaconia and Professional Practice VID Specialized University Oslo Norway; ^2^ Faculty of Health Studies VID Specialized University Oslo Norway; ^3^ Department of Welfare and Participation Western Norway University of Applied Sciences Bergen Norway; ^4^ Department of health and nursing science, Faculty of Health and Sport Sciences University of Agder Kristiansand Norway

## Abstract

**Aims and Objectives:**

To explore the professional practice of transferring patients with lung cancer from hospitals to their homes through the experiences of healthcare professionals (HCPs) working in hospitals.

**Background:**

Hospital‐to‐home transitions are particularly challenging for vulnerable patients, including lung cancer patients, and could threaten patient safety. There is a need to improve coordination between specialised and community care and to develop knowledge on the practice of transferring patients with lung cancer.

**Design:**

A descriptive qualitative design was used. Consolidated criteria for reporting qualitative research (COREQ) were followed for reporting.

**Methods:**

Six focus group interviews with nurses and two focus group interviews with physicians at pulmonary medicine units in two hospitals in Norway were conducted. Qualitative content analysis was used to analyse the focus group interviews.

**Results:**

Patients' vulnerability and gratitude motivated HCPs to ensure that patients experienced the best hospital‐to‐home transition. The following obstacles made it challenging to plan for a good hospital‐to‐home transition and to transfer the responsibility for the patient to the municipality: lack of time and routines to attend to the patient's individual needs, lack of established standards for patient information, absence of resources and predictability, and inadequate communication tools for collaborating with the primary healthcare services.

**Conclusion:**

The healthcare system does not provide hospital units and HCPs with adequate resources to accommodate the individual needs of lung cancer patients in hospital‐to‐home transitions. HCPs compensate with supplementary initiatives to secure patient safety, but the additional responsibility and tasks leave them overworked.

**Relevance to Clinical Practice:**

The study provides knowledge on lung cancer patients' needs in hospital‐to‐home transitions and how HCPs try to ensure patient safety by compensating for the healthcare system's deficiencies.

**Public Contribution:**

A reference group comprising one patient representative from the Norwegian Cancer Society and five HCPs with varied relevant backgrounds contributed to the overall design, recruited participants, and provided feedback on the interview guide.

## 1 Introduction

Transitions between different levels of healthcare services, including both the process and outcome of person–environment interactions, can be challenging for patients (Danielsen et al. 2018; Ellingsen et al. 2014; Flink, Ekstedt, and Flink 2017; Kyte et al. 2019). Transitions from hospital to home can cause significant patient harm (World Health Organization 2016), due to missing information or errors in discharge communication between hospitals and community home care (Connolly et al. 2009; Hesselink et al. 2013; Williams et al. 2015). Thus, transitions can impede healthcare professionals' (HCPs) ability to provide patient‐centred care (Nicosia et al. 2018).

Transitions in healthcare are particularly challenging for vulnerable patients, such as those with low socioeconomic status or poor health literacy (Boyle et al. 2017; Kangovi, Barg, et al. 2014; Kangovi, Levy, et al. 2014). This population has a significant risk of developing psychological problems during transitions (Meleis 2010). Patients with lung cancer are vulnerable (Haas et al. 2018; Lee, Lee, and Chang 2018), having the highest mortality rate among cancer patients worldwide and with an estimated 1.8 million deaths annually. In 2020, lung cancer ranked second among the most commonly diagnosed types of cancer (Sung et al. 2021). Caring for patients with lung cancer arguably has an additional dimension due to the dual stigmas of smoking and poor prognosis, which the literature calls “stacked stigma” (Conlon et al. 2010). Patients stigmatised by society can internalise the stigma and feel responsible for having contracted the disease (Conlon et al. 2010). Among physicians and nurses, lung cancer has been ranked as the type of cancer associated with the lowest prestige (Album and Westin 2007; Johannessen, Album, and Rasmussen 2020).

Ensuring a safe transition for lung cancer patients between different healthcare system levels requires better knowledge of both current challenges and practices. We identify two key sources for the needed insights: the perspectives of HCPs and the structural conditions that govern the medical field. We reached this conclusion by exploring the professional practices and experiences of hospital‐based HCPs who transfer lung cancer patients from hospitals to their homes.

## 2 Background

### 2.1 Lung Cancer Patients and Transitions

Lung cancer patients experience multiple transitions through their illness trajectory. Upon receiving their diagnosis, they mentally change from being healthy to seriously ill. Throughout their treatment and care, they must relate to multiple HCPs at different levels of the healthcare system and transition between their homes and the hospital. Patients with advanced lung cancer experience a high level of distress related to respiratory symptoms (Molassiotis et al. 2010). When patients transition from hospital to home, they leave behind the hospital's safe environment and specialised HCPs. Those who have undergone surgery report significant problems related to the hospital‐to‐home transition, including feeling unprepared, insecure, and unprotected. They may also find postoperative treatment at home challenging due to a lack of information and follow‐up (Kyte et al. 2019; Oksholm, Rustoen, and Ekstedt 2018).

HCPs, especially nurses, play a significant role in facilitating patient's transitions (Meleis 2010), participating in the evaluation of the patient's readiness for discharge, for example. The characteristics of the patient's home also play a vital role in the patient's ability to cope with the transition (Weiss et al. 2007). Notably, when patients return home, there is often a gap in the continuity of care, which could endanger patient safety. Cook, Render, and Woods (2000) argue that while such gaps can never be eliminated, bridges can be built to ensure safer transitions. Thus, the goal is not a seamless hospital‐to‐home transition, but rather a robust and reliable bridge between hospital and community care at home. However, research indicates that existing bridges do not adequately cover the gap and need to be reconstructed. A recent survey found that nurses consider the quality of transitions between different levels in the healthcare system inadequate (Gautun, Bratt, and Billings 2020). Lundereng, Dihle, and Steindal (2020) note that nurses experience early discharge planning as strenuous and excessively time‐consuming. Given that nurses play an essential role in coordinating transitions (Allen 2014, 2019; Kise Hjertstrøm, Obstfelder, and Norbye 2018; Melby, Obstfelder, and Hellesø 2018), these findings are concerning. In addition, studies demonstrate that patients' medical records and other hospital information are often incomplete, resulting in a lack of information on the continuity of care for the patient's general practitioner (GP) or home nursing staff (Flink et al. 2015; Kneck et al. 2019). Additionally, different levels of healthcare commonly utilise different digital platforms for medical records, reducing transparency, sometimes leaving HCPs with missing or conflicting information. Consequently, HCPs provide information based on discretion rather than on established procedures or guidelines (Wibe, Ekstedt, and Hellesø 2015).

### 2.2 Healthcare and Forms of Governance

The New Public Management (NPM) reform in the 1980s shifted public service management towards market‐like principles, aiming to improve quality and efficiency. In many countries, such as the Nordic countries, Portugal, Spain and the United Kingdom, regulation, financing, and provision are now governed by the state, leading hospitals to operate as state‐owned health enterprises (Böhm et al. 2013). Health enterprises lean heavily on market instruments such as cost management, competition, and quality control (Stephansen 2020), making HCPs responsible for both patients' treatment and the state's requirements for efficiency and accountability. In Norway, the state‐owned health enterprise model was introduced in 2002 (NOU 2023: 8). The health enterprises are based on the Nordic welfare state's principle that all citizens must have equal access to healthcare services (Hansen and Dahl 2023). This resembles other welfare systems, such as the UK Healthcare System. However, it has been argued that the Nordic countries might be heading towards a more commercial healthcare system that follows the principle of NPM, which does not align with the values of the Nordic welfare state's principles (Dahlborg et al. 2021).

Improving coordination between specialised and community care has been a core focus in global health policies for the last 20 years (World Health Organization 2016). This approach is also seen in Norwegian health policies (Stephansen 2020). In 2012, Norway implemented the Coordination Reform to provide patients with services from different levels of the healthcare system in more seamless trajectories, accomplished through better cooperation between municipalities and specialised care (Meldt. St. 47 (2008‐2009)). Economic management tools were implemented to reduce the number of hospital admission days by transferring more responsibility for the patient to the municipal level. Therefore, patients discharged from hospitals are on average sicker and require more advanced healthcare, and hospital‐to‐home transitions have become more fragmented (Haukelien, Vika, and Vardheim 2015; The Research Council of Norway 2016). Several efforts have been made to ensure the quality of transitions, e.g. cancer pathway models (The Norwegian Directorate of Health 2021) and a national patient safety program, whose measures strive for a safe patient discharge (The Norwegian Directorate of Health 2017). In addition to the health authorities' guidelines, there are also local procedures and checklists developed by hospitals or hospital wards. All these practice guidelines attempt to standardise patient pathways, assuming all patients have the same needs.

Lung cancer patients are typically not admitted to the hospital inpatient ward for treatment. Instead, they receive treatment at an outpatient clinic at the pulmonary medicine unit, and some patients pair this with follow‐ups by their GP. Due to more accurate diagnostics, treatment and advanced technology, the outpatient treatment model has become an international trend for cancer patients in general in many countries. Typically, patients are admitted to the hospital ward only in the event of post‐surgery treatment, complications during treatment, or at later stages when palliative care becomes necessary. Thus, many patients live at home without daily access to healthcare services. Nevertheless, several develop a need for home‐based care as the cancer develops. The variation of the cancer disease means that these needs vary considerably, and they sometimes remain unmet.

## 3 Method

### 3.1 Study Design

As the first of three sub‐studies exploring hospital‐to‐home transitions for lung cancer patients, this study explores the experiences of hospital‐based HCPs working in specialised care.

A descriptive qualitative design was chosen in order to remain close to the data and avoid adding an interpretative level to the data analysis (Polit and Beck 2020, 486). The data were obtained through semi‐structured focus group interviews. In addition to enabling a researcher to obtain the perspectives of many participants at once, this method increases the probability of creating discussions. Additionally, because HCPs are accustomed to working in teams, the group dynamic can put them at ease, enabling them to share their opinions more easily. An inductive qualitative content analysis inspired by Lindgren, Lundman, and Graneheim (2020) and Bengtsson (2016) was used to analyse the material.

### 3.2 Setting, Recruitment and Participants

Participants were recruited from one university hospital and one local hospital in two different cities in Norway. To recruit participants with substantial experience with lung cancer patients and the hospital‐to‐home transition (Polit and Beck 2020, 499), purpose sampling was used; one contact person from each hospital known to the second author through previous collaborations served as a gatekeeper. The inclusion criteria required that each participant (a) worked as a nurse or physician at a pulmonary medicine unit in a specialised care hospital and (b) had experience caring for lung cancer patients in hospital‐to‐home‐transitions. All physicians were specialists in lung diseases and their work experience ranged from 5 to 32 years, except for one physician specialising in oncology. Some of the nurses had additional education in pulmonary diseases or palliative care. The nurses' experience with lung cancer patients varied from 1 week to 34 years. However, most nurses had worked at their current workplace for several years. Prior to the focus group interviews, the researchers had no contact with the participants, who were unknown to them.

### 3.3 Interview Guide

A semi‐structured interview guide was developed by the authors and discussed with a reference group consisting of one physician who specialised in lung diseases, one GP, one nurse working in a hospital inpatient ward's pulmonary unit, one nurse working in an outpatient clinic's pulmonary unit, one nurse working in home‐based care, and one patient representative from the Norwegian Cancer Society. The group did not suggest any major changes but added some additional details to some questions.

The interview guide contained 11 open‐ended questions (see Table 1). The questions focused on what HCPs do when transferring patients from the inpatient ward or the outpatient clinic to their homes, how the municipal healthcare services collaborate with the patient's GP, what knowledge the patients need, and how HCPs provide information. HCPs were asked to elaborate on potential individual adaptations, the patient's involvement, cooperation with the municipal community services, and preparations in the patient's home before the transfer. The co‐moderator then summarised the interview and asked additional or explanatory questions to the group. Finally, the participants were asked to state what they thought was most important and to mention any relevant detail that could have been overlooked.

**TABLE 1:** Semi‐structured interview guide.

	Questions
1	Presentation round: Please tell us a little about yourself. How long have you worked here, and what are your work tasks? (Introductory questions/icebreaker)
2	What sort of treatment and care do you provide for lung cancer patients? Is there anything special about lung cancer patients or about working with them? If so, please explain
3	Could you please walk me through what you do for lung cancer patients during the hospital‐to‐home transitions? What resources do you need to provide good patient care during a hospital‐to‐home transition?
4	What knowledge does the patient need when transferred to their homes? What information is most important for patients to understand before being transferred to their homes
5	Could you tell me how you provide information to the patient before the hospital‐to‐home transition? How do you determine whether the patient understands and remembers the information provided? How do you involve the patients' relatives when you provide information?
6	Could you share some examples of individual adaptations provided for the patients?
7	How is the patient involved in planning the hospital‐to‐home transition?
8	How do you cooperate with the municipality healthcare services and the patient's GP in hospital‐to‐home transitions? How do you notify the municipality and the GP about the discharge date and how do you communicate information to them?
9	How should the patient's home be organised and prepared before discharge? Who has the responsibility for preparing the patient's home?
10	Is there anything that we have not discussed that you consider essential for lung cancer patients in hospital‐to‐home transitions?
11	What do you believe is the most important topic discussed today?

### 3.4 Data Collection

The gatekeeper from each hospital scheduled the focus group interviews, which were conducted at the hospitals between March and June of 2021; the first author led the interview while one of the other authors served as a co‐moderator. In total, there were eight unique focus groups, six with nurses and two with physicians (Table 2). Each focus group interview included two to five participants; although groups of four to seven had initially been planned, the COVID‐19 pandemic and subsequent restrictions resulted in smaller groups. The gatekeeper had been asked to gather as many participants as possible without interfering with the hospital's daily operation, and the interviews were conducted during the participants shift. No information was collected if a prospective participant refused to participate. Each interview lasted approximately 1 h, was tape recorded, and was then transcribed verbatim by the first author. The transcripts were not returned to the participants for comment.

**TABLE 2:** Participant characteristics: Characteristics of the 28 participants recruited March–June 2021 from two hospitals for eight focus groups.

Focus group interview (*n* = 8)	Participants (*n* = 28)	Professional background and participant code	Department	Setting
1	3 females	Nurses (N1, N2, N3)	Hospital inpatient ward	Pulmonary medicine unit, university hospital
2	3 females	Nurses (N1, N2, N3)		
3	4 females	Nurses (N1, N2, N3, N4)	Outpatient clinic	
4	5 (1 male, 4 females)	Physicians (P1, P2, P3, P4, P5)	Hospital inpatient ward and outpatient clinic	
5	4 females	Nurses (N1, N2, N3, N4)	Hospital inpatient ward and outpatient clinic	Pulmonary medicine unit, local hospital
6	3 females	Nurses (N1, N2, N3)		
7	4 (1 male, 3 females)	Nurses (N1, N2, N3, N4)		
8	2 females	Physicians (P1, P2)		

### 3.5 Data Analyses

The inductive qualitative content analysis of the material was inspired by Lindgren, Lundman, and Graneheim (2020) and Bengtsson (2016). First, all authors read two randomly selected interviews separately and discussed them to acquire an overall understanding of the content related to the aim of the study. Further, all focus group interviews were read by the first, second and last author. Each interview was broken down into smaller meaning units, focusing on identifying content related to the HCPs' experiences of hospital‐to‐home transitions. The condensed meaning was then extracted and assigned a code by the first author. After all the focus group interviews had been coded, the abstracted meaning units and the corresponding codes were reviewed once more before they were divided into subthemes. Each subtheme was then associated with an overall theme. By the completion of that process, it had become evident that the content of the themes would allow their separation into two additional levels. Some themes concerned practices within the hospital unit while others were focused on the system level and external collaboration (see Table 3). Throughout the entire analysis process, all authors participated in the discussion and continually moved between both the descriptive and interpretive levels as well as between the levels of the close or concrete and the distant or abstract (Lindgren, Lundman, and Graneheim 2020).

**TABLE 3:** Content analysis of data transcribed from eight focus group interviews with nurses and physicians (*n* = 28). Examples of meaning units, condensed meaning units, codes, subthemes, themes, and levels.

Meaning unit	Condensed meaning unit	Code	Subtheme	Theme	Level
I tried pretty hard to implement it [a standardised information package for discharge), but it took too much time	I tried to implement a standardised information package for discharge, but it took too much time	Lack of time is a hindrance for quality improvement	Lack of routines for patient information	Lack of time and established standards for patient information	Transition, treatment, and care within the hospital unit
We [physicians] very often find that the GPs withdraw. They leave a lot, everything that comes with it [lung cancer], to the physicians here [at the hospital]	We often experience that the GPs withdraw, leaving everything that has to do with lung cancer to the physicians at the hospital	GPs' follow‐up is lacking	Physicians feel that GPs leave the responsibility for lung cancer patients to them	Struggle to transfer responsibility for the patient	Collaboration between the specialised and primary healthcare services

### 3.6 Rigour

Trustworthiness was a consistent focus. The participants were placed in groups by profession (nurses or physicians) to ensure a comfortable group dynamic and put them at ease in expressing opinions about the topics (Polit and Beck 2020, 515). During each focus group interview, the moderator used member checking to increase credibility (Polit and Beck 2020, 573–575), restating or summarising information and then questioning the participants to determine the accuracy of their understanding.

The five authors have different backgrounds in the previous use of theory, methods, and research areas; the third author is trained in social science and the other authors in nursing. This was considered a strength throughout the process. Each stage of the analysis was performed several times to maintain the quality and reliability of the process. In addition, the Consolidated criteria for reporting qualitative research (COREQ) was used to enhance the quality and transparency of the study (Tong, Sainsbury, and Craig 2007).

### 3.7 Ethical Considerations

Ethical approval was granted by REDACTED (ref. nr. 190602). The Regional Committee for Medical and Health Research Ethics REK Western Norway determined that the project was not subject to submission (ref. nr. 240604). Institutional approval was obtained from the two hospitals included in the study and informed written and oral consent was obtained from all participants before the focus group interviews. The participants were informed that they were free to withdraw from the study at any stage of the interview process without any consequences and that all data would be anonymised. Interview transcripts and audiotapes were kept in separate locked files, and only the authors had access to the data.

## 4 Results

The analysis indicated that all HCPs strove to provide the patient with the best possible hospital‐to‐home transition. Although several barriers obstructed their clinical practice, loyalty to patients motivated the HCPs to overcome the obstacles. Two overarching themes emerged from the data analysis: (1) transition, treatment and care within the hospital unit, and (2) collaboration between the specialised and primary healthcare services (see Table 4).

**TABLE 4:** Results showing overarching themes and sub‐themes.

Overarching themes	Subthemes
Transition, treatment, and care within the hospital unit	Patient vulnerability as a source of motivation for the healthcare professional's work Individual and standardised practices for hospital‐to‐home transitions A lack of time and established standards for patient information An absence of resources and predictability in planning the hospital‐to‐home transition
Collaboration between the specialised and primary healthcare services	The struggle to transfer responsibility for the patient Inadequate communication tools

### 4.1 Transition, Treatment, and Care Within the Hospital Unit

Four themes related to the hospital's organisation of treatment, care, and transition were identified: (1) patient vulnerability as a source of motivation for healthcare professional's work, (2) individual and standardised practices for hospital‐to‐home transitions, (3) ‘a lack of time and established standards for patient information’, and (4) an absence of resources and predictability in planning the hospital‐to‐home transition.

#### 4.1.1 Patient Vulnerability as a Source of Motivation for Healthcare Professional's Work

All the HCPs spoke engagingly when explaining that caring for the patients' well‐being strengthened their commitment to ensuring their needs were met. They described lung cancer patients as a vulnerable group and discussed how they were affected by their vulnerability. Some nurses felt that lung cancer patients sometimes lack access to benefits offered to other cancer patients:

2‐N2: …I have always felt that lung cancer patients are left in the shadow when compared to other cancer patients placed in the cancer ward… Maybe because this is a mixed inpatient ward, we have patients with COPD [chronic obstructive pulmonary disease], asthma, cystic fibrosis, and pneumonia too…Why don't these patients [lung cancer patients] have similar access to benefits as other cancer patients?

They often met patients who blamed themselves for their lung cancer because they had smoked. The HCPs felt obligated to help patients deal with their self‐blame and believed no patient should feel ashamed. Further, several HCPs said that some patients were not only ignorant of their legal rights, but would often not insist on their rights, because they felt guilty. A conversation among physicians exemplifies this:

4‐P1: Lung cancer is so severe that it somehow overshadows everything in life. Everything else becomes less important … I think several of those who have smoked for years feel that it was their own fault [getting cancer].

4‐P4: They do not complain.

4‐P1: No, they feel guilt. And therefore, they feel they cannot be demanding.

4‐P3: And they are grateful for so little, although they are so sick. I agree with you when it comes to the gratitude they show us…We do get criticism, but it is rare.

The HCPs reported that the patients showed gratitude for small gestures, even though they were terminally ill. All HCPs agreed that the patients' gratitude provided meaning and was an important reason to continue their work. One of the nurses described this:

6‐N2: I think they are wonderful, wonderful. I have tried to work in other places, but I just want to go back. They are humble, they are grateful, they are nice, nice people.

#### 4.1.2 Individual and Standardised Practices for Hospital‐To‐Home Transitions

The HCPs indicated that the hospital units have standard routines and checklists for carrying out hospital‐to‐home transitions. However, some HCPs had never used them or were not familiar with their content. In addition, the standard routines were often not used in transitions due to high staff turnover and a lack of time. Consequently, the quality of the transition was greatly affected by the competence, opportunity, and motivation of the particular HCP involved. This was explained by a nurse:

7‐N4: I tried to introduce it [the routine for transitions], and it went well for a while.

7‐N3: Yes, it did.

7‐N4: But there is always something that slips, an obstacle that's always there; it takes too much time. The inpatient ward is a high‐turnover ward. We have a permanent team [of staff] that works here, and then we have those who drop in … They destroy the rhythm.

The HCPs emphasised that the great variation in individual needs was an obstacle to implementing standardised procedures for hospital‐to‐home transitions. Further, lung cancer patients were not discharged just once; they often went through multiple transitions between the specialised care facility and their home. As seriously ill patients with a short life expectancy, lung cancer patients have different needs that depend on how advanced and prolonged the disease is. The time spent in specialised care is an intense period, typically lasting months or years but sometimes only weeks. A conversation among physicians illustrate these challenges:

4‐P1: It's often hard to map out what they need in their homes […] they say they do not need anything, but then you see that there are most likely some needs.

4‐P2: And we tell them they must contact the cancer coordinator, and some do. There are good experiences and bad experiences.

4‐P1: At the outpatient clinic, we have the entire spectrum of patients. Some you call the day before treatment and ask: "How are you?" [and the patient replies,] “It's going well. I'm skiing at the moment”. And then you have the next one, and it's like “Hello? [Imitates a weak voice]. No, I do not know if I can come, I have to have an ambulance.”…. The patients are very different. Some live for many years and live almost as normal, while others have a significant symptom burden from the very beginning. So, it's not one standard for all.

4‐P5: That's true, because when you discharge someone from the hospital where you know they are going home to spend their last days, you are more systematically…almost as if you create a miniature hospital at their homes.

In the absence of a standardised practice, HCPs took the initiative to improve the transition. For example, nurses in the outpatient clinics contacted patients between appointments to follow up on matters that were not strictly the hospital's responsibility. Sometimes, they allowed patients who felt unsafe going home to stay an additional day in the hospital.

#### 4.1.3 A Lack of Time and Established Standards for Patient Information

All HCPs stressed the importance of ensuring that patients understood and remembered the information provided. However, neither the nurses nor the physicians said they had any ‘information package’ to patients on their way home. The HCPs provided many examples of measures to help patients remember information. For example, one physician said she always wrote notes for the patients in addition to giving them verbal information to ensure that they would not forget. The physicians always consulted with their patients before discharge, providing information supposedly based on the patient's individual needs. However, the patient's individual needs had rarely been adequately identified beforehand. Some physicians acknowledged that these consultations could be challenging due to an overloaded schedule and a lack of procedures for providing information. Both hospitals had a routine for the physicians' written summary of the hospital stay. Although this summary note is intended to be part of a dialogue between the patient and physician before discharge, the dialogue seldom takes place, as this conversation among nurses illustrates:

5‐N4: I often think, you know what, my experience is that the physician arrives in the morning and says [to the patient], "Yes, today you will be discharged. I will write you a written note with information and return to you with that". However, I often experience that the physicians have so much to do that they hand the information to us and say, "Can you just give this to the patient?". And then it will be like, "Here's the note from the physician." [The patient replies,] "Oh, but the physician said that he would come back."

5‐N3: It happens.

5‐N4: It actually happens quite often, and I get pretty embarrassed. It is an alarming trend. Many physicians do that.

5‐N1: Yes, in that way, the first patient round gets done quickly because they say they will come back.

5‐N4: And they often say, "I will look at your bloodwork and your medications and return with that note." And then the patients don't get to know the answers to the bloodwork, and maybe they have gathered up some questions as well, but everything goes so fast. Instead, the nurse returns with the physician's note and says, "We have ordered you a taxi to take you home."

Nurses and physicians considered the patients' relatives to be vital in assisting patients to remember information provided by the HCPs and frequently included them when providing information. The HCPs also explained that many lung cancer patients are accustomed to managing on their own. One nurse at the local hospital described the patients as “Lonely cowboys who have lived a rough life and do not have so many loved ones close around them” (6‐N2). Unless a relative was present, patients often did not understand the information they were given, as some of the physicians explained:

4‐P4: I almost force them to bring relatives with them, and I have done that throughout COVID‐19.

4‐P2: Yes, that is important.

4‐P5: I had one patient yesterday who could not remember anything because of head trauma many years ago. So, I tried to provide information from her husband, but he was over 85 years [old] and did not remember much either. So, I told them to bring their son the next time, so I know what I say will be remembered.

4‐P1: It is incredible how little they remember and how they focus on the wrong things if you ask them to summarise what you said. And the discharge summary says in black and white what kind of information they have received, and it can be something completely different that comes up. And they do not even have dementia, but they may, in a way, not have caught up with what is happening.

The patient information the nurses provided from the inpatient rounds was often based on the physician's information, simplifying and explaining the physician's words without adding anything. While the patient information provided by the physicians was thorough, the patients often did not understand it. Nearly all the nurses working in the hospital inpatient wards joined the physicians during patient rounds so that later, they could explain the information again in more straightforward language. Some nurses and physicians admitted they could have used techniques such as the teach‐back method more often to ensure that the patients had understood. Here too, they cited the lack of established routines as the reason they often forgot to make time. However, the nurses in the outpatient clinic provided the patients with standard information about their treatment.

#### 4.1.4 An Absence of Resources and Predictability in Planning the Hospital‐to‐Home Transition

According to the HCPs, a hospital‐to‐home transition is very challenging logistically. In an ideal case, discharge planning should begin when the patient is admitted to the hospital. Often, however, that did not happen. Decisions about discharges were frequently made suddenly and often on Fridays to ease the weekend workload. If the discharge plan had not been prepared, the transition could be suboptimal. Sudden discharge decisions tended to result in incomplete information and a transition that was stressful for both the patient and the HCP. One physician shared this personal experience:

4‐P1: It is good if the discharge note is prepared. I was working this weekend, and there were several phone calls and messages from the home care nurse because the physician on call before the weekend had been so busy. He had not written prescriptions and had sent the patient home with a patient orientation note and some pills in a bag. So, that was a bit deficient. I ended up writing prescriptions to avoid them [home care nurses] readmitting him in pure protest.

The nurses were responsible for planning hospital‐to‐home transitions. Effective planning depended on well‐functioning interdisciplinary cooperation within the hospital. However, several positions in the pulmonary medicine units had been eliminated, leaving fewer staff to shoulder the same workload. The nurses feared they might be less able to determine what a patient would need from the municipal healthcare services after discharge or how the municipal services should meet those needs. A conversation among three nurses revealed that their care for an individual patient was being compromised by their many tasks:

1‐N1: We often get questions from the home nursing staff or the coordinator [of healthcare services] in the municipalities about the patient's ADL functions [activities of daily life]. We are a busy ward, so it is not always we have time to….

1‐N2: To perform a patient ADL checklist.

1‐N3: That is right. Because in a way, we do not have time to be that close to the patient.

1‐N2: We provide treatment. That is antibiotics, painkillers, intravenous fluids, take vitals and stuff like that.

All HCPs stated that they depended on relatives to help identify the healthcare services the patient would need at home. The patient often does not fully comprehend their situation and may struggle to realise that they need more help than their relatives could provide.

### 4.2 Collaboration Between the Specialised and Primary Healthcare Services

According to all HCPs, the overall primary and specialised healthcare systems regulate the organisation of the pulmonary medicine unit. To provide the patient with a safe, quality‐assured hospital‐to‐home transition, HCPs cooperated with partners in the municipality using digital platforms. Two themes were identified for this system‐level organisation: (1) the struggle to transfer responsibility for the patient and (2) inadequate communication tools.

#### 4.2.1 The Struggle to Transfer Responsibility for the Patient

The physicians stated that they followed patients throughout their treatment trajectory. When asked at what point a patient is no longer the responsibility of the specialised care, they all answered that this responsibility rarely ends. They wanted patients to have access to the health services they need at all times, and therefore, they circumvent the system when necessary to ensure this. Some physicians arranged fictional appointments at the outpatient clinic to ensure that the patient had an open course of treatment with their primary hospital physician, even if the patient's treatment had ended, or if they expected the patient to be deceased by that time. Although the patient's GP often had medical responsibility, the physicians explained the patient often preferred help from a hospital physician. They wanted to guarantee the patient easy access to specialised care and contact with a physician they trusted, in case the GP's help was inadequate. An open course of treatment did not require a new referral from the GP, and it enabled the patient to contact the hospital independently and immediately if they needed help. Most importantly, the physicians explained that they did this because they had become attached to the patient:

4‐P4: And I think it helps that we follow them. We perform the medical investigation, and we follow them until they die. We have the entire patient trajectory. There are many [patients] for whom I have performed their first biopsy and later also written the death certificate. And then it's like that; if you cannot give them cancer treatment, then you can always give morphine or sort out palliative needs.

4‐P3: But maybe that is why we take some ownership of them?

P4: Yeah.

4‐P3: Yeah, and the GPs are out there…. It is a bit challenging. I love our patients, and we develop a relationship. So, when it comes to the end [death], patients have often become attached to us and the nurses.

The physicians often felt that the GPs did not take responsibility for care between appointments or after treatment had finished, and that led the physicians to take over the GPs' tasks. The reason the GPs hesitated to take responsibility, the physicians believed, was that they were afraid of making mistakes. Therefore, they reasoned, the GPs would be more likely to assume their responsibilities if they were more involved in the patient's treatment trajectory from the beginning. One physician emphasised a desire to involve the GPs more:

4‐P3: We have tried to invite them [the GPs] to collaboration meetings inside the house [the hospital]. But they are totally overworked; it is impossible to make it happen.

The HCPs explained how healthcare services provided for cancer patients varied across municipalities according to local capacities and competencies. These variations undermined the possibility of a standard procedure for the hospital‐to‐home transition; although the same equal minimum standard applied for all patients, the specialised care unit could not follow the same procedure or expect the same service from all municipalities. For example, some municipalities did not perform home visits to evaluate the patient's needs, whereas others offered extensive services, such as physiotherapy, psychology, or social activities for cancer patients. Municipalities also differed in whether they had a local coordinator for cancer patients to provide an overview of the available services and assistance. The HCPs argued that a local coordinator for cancer treatment was essential for patients who did not have relatives to help them.

#### 4.2.2 Inadequate Communication Tools

The participants described how the specialised and municipal healthcare services communicated with one another digitally, by phone and sometimes by letter to coordinate patient trajectories and manage collaborative relationships. They preferred electronic messengers over telephone communication, although the current digital system had flaws. Specifically, they complained that it has not kept pace with developments in specialised treatment, specifically with the shift to treatment at outpatient clinics. Electronic messages still coordinate the patient's care only between hospitalisations, whereas coordination is also needed between treatments. This was discussed by nurses from the outpatient clinic.

3‐N3: These electronic messages…that's the first thing that strikes me as a good tool. The inpatient wards have access, but we don't.

3‐N4: The physicians can send them, but then we have to nag at them. And the physicians write differently than us. We see other things…We can phone them [nurses in community care], but that is a barrier because we have to find out which district they belong to, who's there, or maybe we have to write a letter.

3‐N1: If we had the opportunity [to write an electronic message to the community care] it would be so much easier.

3‐N3: It would have saved us for incoming calls too. Because there are also nurses in home‐based care who call us, and it disturbs our work.

3‐N2: I'm sure that we would have contacted them earlier if we had the opportunity to use electronic messages.

Both nurses and physicians working in the outpatient clinic were dissatisfied that only the physicians could send electronic messages to the community care services and to the patient's GP. If the nurses in the outpatient clinic wanted to communicate with the community care services or the GP, they had to use the phone. However, when the nurses and the primary healthcare facility communicated by telephone, there was the risk that important details could get lost, which posed a risk to patient safety. In addition, the outpatient clinic's nurses admitted applying for community care services too late in the patient's treatment trajectory. One nurse in the outpatient clinic described the system's complexity:

5‐N4: Just the other day, Monday, I had a patient who needed community care services… I called the municipality and said that I would like to apply for home‐based nursing for her, and they answered “No, you cannot do that. The patient must apply herself, either electronically or in writing.” So, then I printed it out [the application form] because it is not easy for these elderly people to do it.

The nurses also complained that if the patient applied for home‐based services without having been hospitalised, it could take weeks before a representative from the home‐based office visited the patient's home. Yet, this was senseless because if the patient was admitted to the hospital's inpatient ward, the healthcare services would be in place the same or the next day after the patient was discharged. This is because the nurses in the hospital's inpatient ward have access to the digital system and can apply for home care services directly.

## 5 Discussion

The results of this study show that a lack of resources and standard procedures impede HCPs' efforts to meet the needs of lung cancer patients during hospital‐to‐home transitions. In addition, the conditions for cooperation between the specialist and municipality healthcare services are inadequate. Due to their strong engagement and commitment to lung cancer patients, HCPs compensate for the system's flaws through discretional initiatives. This leads HCPs to take on additional responsibility and tasks, leaving them overworked. Throughout a patient's trajectory, the interests of the healthcare system as a whole conflict with those of HCPs, who focus on trying to meet the needs of patients. The discussion below addresses that conflict, illuminating how and why HCPs undertake compensatory practices. Although this study was conducted in a Norwegian context, the results are transferable to other countries with a similar national healthcare system run and financed by the state, such as the other Nordic countries, Portugal, Spain and the United Kingdom, as the findings address challenges based on the system's structure.

### 5.1 Conflicting Interests

In principle, a patient's needs should be met through the frameworks provided by the health authorities, such as different policy reforms and their distribution of responsibility or guidelines from clinical pathway models. However, the findings of this study nuance this assumption. Confirming research by Veenstra and Gautun (2021), we found that focusing on clinical pathway models for specific diseases during hospital‐to‐home transitions obscures patients' needs in their homes after discharge. This happens because the pathway models focus primarily on the disease itself instead of the patient's needs. In addition, lung cancer pathways can be complicated by a number of factors, including limited resources, the HCPs' increased workload without compensatory resources, local differences and complex needs of patients (Otty et al. 2020). The HCPs in our study portrayed lung cancer patients as a complex, varied and vulnerable patient group, each requiring individually adjusted care. Consequently, standardised checklists or procedures for hospital‐to‐home transitions cannot effectively define or predict their individual needs. When standardised checklists and procedures are applied, HCPs are left with the responsibility to provide compensatory initiatives to ensure that patients' individual needs are met.

Some argue that Norway weakened professional and clinical autonomy by implementing an organisational model of health enterprises in 2002, situated within the wider framework of NPM (Stephansen 2020). This is not unique to Norway. Similar critiques have also been raised towards other healthcare systems, such as in the UK and Finland, where the impact of NPM policies has been claimed to weaken professional ethics and endanger the trust relationship between professionals and their patients (Siltala 2013; Simonet 2013). The findings of this study support these arguments. HCPs clearly indicated that their medical expertise and experience guide them in meeting patients' needs, but system requirements often hinder their efforts to act on their discretionary insights. We interpret the professional practice of the HCPs in our study to be based on a genuine desire to help the patients, thus, a motivation expressed by Larsen (2009, 43): ‘Everything we do, we do to prevent illness and improve health’. This principle is contrary to the values of profit and efficiency represented by health enterprises. In Denmark, it has been argued that implementing quantitative measures to ensure high health quality has exceeded the Hippocratic Oath of HCP, leaving the efficiency and productivity elements from NPM to triumph (Malmmose 2015). Further, it has also been argued that Norway has allowed financial incentives to take precedence over professional assessments in transitions between hospitals and municipalities (Otterstad 2009). As several authors have argued, the state's political power over the health sector has increased. The top management in hospitals has assumed an important role as a caretaker of the state's interests, replacing medical professionals for decisions about the distribution of resources and priorities within the hospital (Byrkjeflot and Neby 2008; Stephansen 2020). The ensuing conflict was inevitable because the bureaucratic field's logic of priorities and the medical field's logic of caring are incompatible.

Still, although HCPs may disagree with health authorities' priorities and distribution of resources, our findings indicate that they are generally compliant with them. Many tasks within the framework of hospital‐to‐home transitions, for instance, include procedures with accompanying deadlines. The hospital management keeps statistics about deadlines, ensuring they are prioritised by the HCPs. According to our participants, the electronic messengers that HCPs use to communicate with the municipalities are inadequate to secure good information flow, and are not adequately adapted for patients receiving treatment in the outpatient clinic; as supported by of Danielsen and Fjær (2010) and Melby, Brattheim, and Hellesø (2015). Further, this illustrates how medical advances have changed the way in which patients are treated. Although intended to be efficient, many guidelines and procedures are misaligned with those medical developments. Therefore, we argue that the HCPs in this study are left with the responsibility to compensate for insufficiencies to ensure patient safety.

### 5.2 Compensating Practices

The HCPs in this study reported time constraints, a lack of resources, and a generally challenging working environment, amplifying the need for compensatory practices. In addition, when physicians felt obligated to follow patients throughout their trajectory, they compensated for flaws in the community care services by undertaking tasks properly belonging to the municipality or the patient's GP. Previous studies on transitions from hospitals to municipalities also found that HCPs compensate for deficient systems, and that their competence and individual actions are essential for ensuring patient safety (Danielsen and Fjær 2010; Sogstad and Skinner 2020). Admittedly, if each HCP compensated for systemic insufficiencies based on their discretional assessment, that could lead to inequitable healthcare services. In addition, the HCPs in this study also indicated that high turnover in the hospital inpatient wards exacerbated the challenges of maintaining procedures for transitions. Consequently, inexperienced HCPs can be left with an overwhelming responsibility. While HCPs stated that lung cancer patients require an individualised approach, they acknowledged that checklists could sometimes be helpful in a hectic work environment. Additionally, it must be acknowledged that patients with lung cancer represent a particularly complicated patient group and may require more resources than other cancer patients.

Importantly, the HCPs stated that their compensatory practices provide vital support for patients to manage the hospital‐to‐home transitions. In addition, they expressed an assumption that patients often struggle to understand the information they receive. Their assumption is supported by research indicating many lung cancer patients have low health literacy levels (Haas et al. 2018; Lee, Lee, and Chang 2018) and little understanding of their disease (Jenum and Pettersen 2014). Thus, as argued by Weiss et al. (2007), it is important that HCPs have skills in delivering information and assessing what content to include, as this affects how prepared patients feel when they are transferred. Based on this study's findings, we argue that lung cancer patients' needs are so individual that standardised procedures for hospital‐to‐home transitions might not be sufficient as they require an individual approach from HCPs. Alternatively, standardised procedures could be supplemented with other individualised approaches to compensate for the gap between unique needs and standardised procedures.

In addition to the HCPs' compensatory practices, the patients' relatives play an essential role in ensuring the safety and quality of their hospital‐to‐home transitions (Lilleheie et al. 2019; World Health Organization 2016). HCPs in this study explained that they relied on patients' relatives when providing information or identifying needs. We argue that the way in which relatives support the HCPs can be considered an additional compensatory practice initiated by HCPs because relatives are expected to assume specific responsibilities to help the patient, relieving HCPs of some of the burden of compensating for systemic shortcomings. Consequently, the relatives' participation in the discharge process helps to ensure that the patient's needs are met. However, this is an unreliable practice. Some patients have relatives who are incapable or would be overwhelmed by becoming informal caregivers (Lilleheie et al. 2021; Rustad et al. 2017), while others have no relatives.

As a preliminary conclusion, our findings indicate that hospitals depend on the commitment of HCPs and what appears to be a form of ‘volunteer culture’. However, the HCPs' commitment and volunteer work remain unacknowledged, nor is it measured or recognised by the hospital management or the health authorities.

### 5.3 The Structure Behind the Compensation Practices

By compensating for the system's flaws, HCPs also contribute to maintain the healthcare system's shortcomings; their voluntary work allows the need to improve practices to remain a low priority. We argue that standardised procedures in hospital‐to‐home transitions challenge HCPs' ability to care for lung cancer patients based on their individual needs, creating a dilemma about what to prioritise. As argued, the health authorities within the bureaucratic field establish the framework for healthcare services through regulations and distribution of resources, and their motives and objectives differ from those of HCPs. The medical field and the bureaucratic field operate with different logics and priorities, which lead to different practices. These different logics can be explained using Bourdieu's theory on doxa. Doxa is the dominant consensus regarding what is considered the right practice within a field. It is hidden and yet, so obvious that it is simultaneously invisible and taken for granted by the field's members (Bourdieu and Wacquant 1992). According to Bourdieu, any given field produces such uncontested doxical notions about the ‘correct’ ways of seeing, believing, and acting. Based on our findings, one might argue that the doxa of the medical field is threatened or infiltrated by the bureaucratic field, where efficiency and profit are prioritised above patients' individual needs. For example, by organising patient trajectories through cancer pathway programs, health authorities facilitate and realise an ideology that supposes all patients require the same care. In contrast, the medical field places a high value on patient‐centred care. Although clinical pathway models for lung cancer have been shown to reduce the time needed for diagnosis and treatment (Otty et al. 2020), all HCPs in this study argued that healthcare services must be adapted to each patient's needs. We argue that the current doxa of the medical field is threatened by the bureaucratic doxa, which is based on two conflicting logics: health and economics. Further, this conflicting understanding of reality contributes to the compensatory practices performed by the HCPs. HCP statements indicate that it is both accepted and expected that HCPs should provide more than the system to ensure patient safety and satisfaction. This expectation that HCPs will compensate for the system's deficiencies can be understood as part of the ‘infiltration’ of the bureaucratic doxa.

### 5.4 Strength and Limitations

This study has strengths and limitations. The researchers have no information on how many participants were invited by the gatekeeper compared to how many were recruited. There are also more females compared to males in the sample, thus there is a preponderance of female HCPs. In addition, the economics and resources of hospitals may vary, which could also affect the representativeness of the study sample. The use of focus groups also involves limitations. Some participants may feel intimidated by more experienced and dominant group members, and if they are too hesitant to share their experiences, that will reduce the generalisability of the results. In addition, the first author, who conducted the focus group interviews, was a PhD candidate without experience conducting focus group interviews. Nevertheless, all co‐moderators were experienced senior researchers with substantial previous experience with that type of interview. The patient representative, unfortunately, had to withdraw from the role 1 year into the study due to illness; therefore, the patient representative contributed only to the planning of the study and the development of the interview guide, not to the findings or the revision of the manuscript, which may be a limitation.

## 6 Conclusion

This study shows that the healthcare system does not provide HCPs with adequate resources to accommodate the individual needs of lung cancer patients in hospital‐to‐home transitions. Consequently, HCPs compensate through supplementary initiatives to ensure patient safety and what they believe is in the patient's best interests. They are motivated to do this because of their commitment to the patients. We interpret their compensatory practices as a result of a battle within the medical field about the ‘right’ practice. As long as HCPs' values and competence in caring for lung cancer patients are deprioritised, patients will remain at risk of receiving less individualised healthcare services. In addition, the compensatory practices described in this study are not sustainable in the long term and could lead to HCPs being overworked, which could threaten patient safety. Further research should consider the unspoken expectations of HCPs to help increase awareness of the specific changes needed to improve the quality of hospital‐to‐home transitions. In addition, there is also a need for more research exploring solutions to the challenges that HCPs experience.

## 7 Relevance to Clinical Practice

This study provides important insights into the practice of hospital‐to‐home transitions for lung cancer patients from the view of nurses and physicians working in specialised care. The results offer substantial information about how HCPs address and evaluate lung cancer patients' needs in hospital‐to‐home transitions. In addition, the findings demonstrate that when the healthcare system fails to meet the patient's needs, HCPs ensure patient safety through compensatory individual actions. Recognising HCPs' motivations to care and take responsibility for this patient group offers a good starting point for developing and implementing new measures to improve hospital‐to‐home transitions.

## 8 What Does This Paper Contribute to the Wider Global Clinical Community?

- Hospital‐to‐home transitions are challenging. Several studies suggest different approaches to bridge the gap in transitions. However, research has shown varying results, and studies conducted from a meta‐perspective may be necessary to improve hospital‐to‐home transitions.
- Lung cancer patients are portrayed by HCPs as a complex, varied and vulnerable patient group, with each patient requiring individual hospital‐to‐home transitions. Consequently, standardised pathway models and guidelines may be inadequate to ensure that lung cancer patients' needs are met.
- Healthcare professionals compensate for the system's shortcomings through individual practices based on their professional assessment of the patient's needs. Without these practices, patient safety would be at risk.

## Author Contributions

**Charlotte Nilsen:** conceptualization, data curation, formal analysis, funding acquisition, investigation, methodology, project administration, resources, software, visualisation, writing – original draft preparation (lead) validation, writing – review and editing (equal). **Trine Oksholm:** conceptualization, data curation, formal analysis, investigation, methodology, visualisation, writing – original draft preparation (supporting), supervision, validation, writing – review and editing (equal). **Gudmund Ågotnes:** conceptualization, methodology, visualisation, writing – original draft preparation (supporting) validation, writing – review and editing (equal). **Gro Beate Samdal:** conceptualization, investigation, visualisation, writing – original draft preparation (supporting) validation, writing – review and editing (equal). **Sidsel Ellingsen:** conceptualization, investigation, methodology, visualisation, writing – original draft preparation (supporting), supervision, validation, writing – review and editing (equal).

## Ethics Statement

Any data utilised in the submitted manuscript have been lawfully acquired in accordance with The Nagoya Protocol on Access to Genetic Resources and the Fair and Equitable Sharing of Benefits Arising from Their Utilisation to the Convention on Biological Diversity. The relevant fieldwork permission was obtained from the hospitals included in the study. Ethical approval for this study was given by the Norwegian Agency for Shared Services in Education and Research (ref.nr. 190602). The Regional Committee for Medical and Health Research Ethics REK Western Norway assessed that the project was not subject to submission to them (ref. nr. 240604).

## Conflicts of Interest

The authors declare no conflicts of interest.

## Data Availability

The data that support the findings of this study are not publicly available due to ethical restrictions. However, the data supporting this study's findings could be made available from the corresponding author upon reasonable request.
